# Time to first antenatal care visit among pregnant women in Ethiopia: secondary analysis of EDHS 2016; application of AFT shared frailty models

**DOI:** 10.1186/s13690-021-00720-2

**Published:** 2021-11-09

**Authors:** Kenaw Derebe Fentaw, Setegn Muche Fenta, Hailegebrael Birhan Biresaw, Solomon Sisay Mulugeta

**Affiliations:** Department of Statistics, Debre Tabor University, Debre Tabor, Ethiopia

**Keywords:** Acceleration failure time, Frailty, Inverse Gaussian shared frailty, ANC, Visit

## Abstract

**Background:**

The survival of pregnant women is one of great interest of the world and especially to a developing country like Ethiopia which had the highest maternal mortality ratios in the world due to low utilization of maternal health services including antenatal care (ANC). Survival analysis is a statistical method for data analysis where the outcome variable of interest is the time to occurrence of an event. This study demonstrates the applications of the Accelerated Failure Time (AFT) model with gamma and inverse Gaussian frailty distributions to estimate the effect of different factors on time to first ANC visit of pregnant women in Ethiopia.

**Methods:**

This study was conducted by using 2016 EDHS data about factors associated with the time to first ANC visit of pregnant women in Ethiopia. A total of 4328 women from nine regions and two city administrations whose age group between 15 and 49 years were included in the study AFT models with gamma and inverse Gaussian frailty distributions have been compared using Akaike Information Criterion (AIC) and Bayesian Information Criterion (BIC) to select the best model.

**Results:**

The factors residence, media exposure, wealth index, education level of women, education level of husband and husband occupation are found to be statistically significant (*P*-value < 0.05) for the survival time of time to first ANC visit of pregnant women in Ethiopia. Inverse Gaussian shared frailty model with Weibull as baseline distribution is found to be the best model for the time to first ANC visit of pregnant women in Ethiopia. The model also reflected there is strong evidence of the high degree of heterogeneity between regions of pregnant women for the time to first ANC visit.

**Conclusion:**

The median time of the first ANC visit for pregnant women was 5 months. From different candidate models, Inverse Gaussian shared frailty model with Weibull baseline is an appropriate approach for analyzing time to first ANC visit of pregnant women data than without frailty model. It is essential that maternal and child health policies and strategies better target women’s development and design and implement interventions aimed at increasing the timely activation of prenatal care by pregnant women. The researchers also recommend using more powerful designs (such as cohorts) for the research to establish timeliness and reduce death.

## Background

Antenatal care is pregnancy-related essential health care, which could be given either in a health facility or at home and it is an integral component of maternal and child health. A woman who starts antenatal care at a gestational age of fewer than 12 weeks is referred to as ‘early antenatal care’ [1].

In 2017, almost 295,000 women died from preventable causes related to pregnancy and childbirth around the world, and 810 women died every day. The vast majority of these deaths (94%) occurred in low-resource areas, and many could have been avoided. In the same year, maternal deaths in Sub-Saharan Africa accounted for over two-thirds (196,000) of all maternal deaths, whereas maternal deaths in Southern Asia accounted for nearly one-fifth of all maternal fatalities [2]. It covers a wide range of countries, from industrialized (98%) to low-income (68%). Even though ANC services are becoming more widely available in many African countries, coverage alone does not provide enough information about the service [3].

The global maternal mortality ratio (MMR), or the number of maternal deaths per 100,000 live births, was estimated to be 216, with nearly all (95%) occurring in developing nations [4]. Ethiopia has one of the world’s highest maternal mortality rates, with 412 per 100,000 live births in 2016 [5].

In nations with high maternal morbidity and mortality, timely ANC initiation is critical to reducing maternal morbidity and mortality [6]. Maternal morbidity and mortality were high in developing nations, especially in Sub-Saharan Africa. Most women, on the other hand, started their first ANC in the third or fourth trimester of their pregnancy [4, 6]. According to research conducted in Uganda and Kenya, the first ANC visit took an average of 7 and 5 months, respectively [7, 8]. In a separate study conducted in Northwest Ethiopia, 52% of women scheduled their first ANC appointment after 4 months of pregnancy [9]. .Different studies have been done in the case of identifying risk factors for time to first antenatal care visit among pregnant women in Ethiopia [9–13]. Those studies were done using semi-parametric survival models (Cox Proportional Hazards) and parametric survival models (Exponential, Weibull, Log-normal, Log logistic, Generalized Gamma distributions). However, the studies are not considering the random effects in the model to account for association and unobserved heterogeneity (frailty). Heterogeneity between individuals should be considered when survival data comes from different groups or if individuals have repeated measurements. If heterogeneity is omitted several problems may occur such as overestimation of relative hazard rate, biased estimates of regression coefficients, and making the regression parameters estimate tend to zero [14]. Frailty provides a more precise estimate of parameters compared to standard AFT models [15].

This study used AFT shared frailty to overcome these limitations and to further estimate the significant impact of predictor variables in Ethiopia. Furthermore, to be represented at the national level, the majority of the research is limited to certain districts. As a result, it will be critical for improving the health of newborns, pregnant women, and families, as well as increasing knowledge about maternal and child morbidity and mortality in society. It will also guide policymakers and program planners on the reduction of maternal and child mortality by considering the time in addition to the number of ANC visits at pregnancy. Therefore, this study was designed to identify determinant factors for time-to-first ANC visit using AFT shared frailty models by considering different baseline distributions to investigate a model that is better in predicting time to first ANC visit in Ethiopia.

## Methods

### Study area, study design, and population

#### Study area

This study was carried out in Ethiopia, and Ethiopia was the second-most populous country in Africa next to Nigeria and found in the horn of Africa. The administrative structure of Ethiopia consists of nine regional states (Tigray, Afar, Amhara, Oromiya, Somali, Benishangul Gumuz, Southern Nations Nationalities and People (SNNP), Gambela, and Harari) and two city administrations (Addis Ababa and Dire Dawa) [16].

#### Study design

**Fig. 1:**
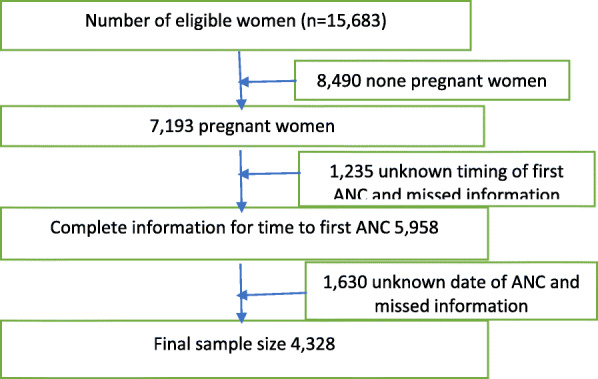
Sample size and sampling procedure to reach the final sample size in 2016 EDHS

We have used 2016 EDHS data. This is the fourth national representative survey done at the country level. The main goal of this dataset was to provide up-to-date information about the key demographic and health indicators. The 2016 EDHS used a two-stage stratified sampling design to select households. In the first stage, there were 645 enumeration areas (202 urban and 443 rural) based on the 2007 Ethiopia Population and Housing Census. A total of 18,008 households were considered, of which 16,650 households and 15,683 women were eligible. The women were interviewed by trained lay interviewers. All women of reproductive age (15 to 49 years) who were either permanent residents of the selected households or visitors who stayed in the selected household the night before the survey, were eligible for the study. A total of 15,683 women aged 15–49 years were interviewed with a response rate of 95% [17]. For the current study 4328 pregnant women from nine regions and two city administrations were included (Fig. 1).

#### Study population

This study was conducted on pregnant women ages 15–49 years from nine regions and two city administrations in Ethiopia by a survey done obtained from EDHS 2016. A secondary data source from 2016 EDHS was used.

#### Inclusion and exclusion criteria

Pregnant women of age 15–49 years and whose gestational age (duration of pregnancy in weeks) was known at first ANC visit were included in the study (event). In addition, women who did not access ANC throughout pregnancy and the duration of pregnancy were recorded at delivery, or termination of pregnancy recorded was also included as censored observation. However, women who had ANC visit but their gestational at ANC visits was unknown (unrecorded) were excluded from this study.

### Variables under investigation

#### Response variables

The dependent variable is time-to-first ANC receipt among pregnant women which is measured in months. The survival time was the duration of pregnancy (in months) measured from the time of conception to the first ANC visit (event) and others who did not attend ANC throughout of pregnancy period regardless of the outcome of pregnancy were considered as (censored).

#### Explanatory variables

Different covariates were considered in this study to determine factors associated with time to first ANC visit. The region was considered as a clustering effect in all frailty models (Table 1).

**Table 1:** List of predictor variables for the assessment of time to first ANC visit in Ethiopia

Variables	Descriptions
Mother occupation	Yes, No
Region	Tigray, Afar, Amhara, Oromia, Somali, Southern Nations Nationalities and People (SNNP), Benishangul-Gumuz, Gambela, Harari, Addis Ababa, and Dire Dawa are categories of the region.
Woman’s age	The age’s of pregnant women was classified as 15–24,25–34, 35–49
Parity	Number of children ever born including the current pregnancy categorized as 3 or less and 4 or more
Media exposure	No, Yes
Women’s Education level	The level of education women attained with categories: No education, Primary, Secondary or higher
Husband Education level	The level of education women attained with categories: No education, Primary, Secondary or higher
Residence	Women’s residence place was Urban or Rural
Wealth index	It was categorized as Poor, Middle, and Rich
Head of a household	Gender of household categorized as male or female
A person who usually decides on respondent’s health care	A person who usually decides on respondent’s health care in the family was coded as Respondent alone, Respondent and husband/partner, Respondent and another person, Husband/partner alone, Husband/partner alone, Someone else, and Other
Marital Status	Separated, Married
Wanted pregnancy	Whether the current pregnancy was wanted then, later and no more
Religion	The religion of pregnant women was categorized as Muslim, Protestant, Orthodox, and Others

### Definition of technical terms

#### Time

Time was measured in a month(s) from date of pregnancy to first ANC booking for women’s having at least one ANC visit and their current gestational age otherwise.

#### Event

The event was considered to happen if the pregnant women had at least one ANC ad considered censored otherwise.

#### Survival analysis

Survival analysis is a collection of statistical procedures for data analysis for which the outcome variable of interest is time until an event occurs.

#### Cox proportional Hazard model

The Cox Proportional Hazard Model is a multiple regression method used to evaluate the effect of multiple covariates on survival time.

#### Accelerated failure time

The accelerated failure time model is an alternative to Cox PH and parametric models for the analysis of survival time data. Unlike the proportional hazards model, it is used to measure the direct effect of the explanatory variables on the survival time instead of a hazard.

#### Frailty

frailty is an unobserved random factor that modifies multiplicatively the hazard function of an individual or cluster of individuals in time to event data [18].

### Statistical analysis

The data set was downloaded from the website https://dhsprogram.com after an approval letter for use had been obtained from the measure DHS. Variables were extracted from the EDHS 2016 kids and individual women’s data set using a data extraction tool. After data management, cleaning and weighting descriptive measures such as median, percentage, graphs, and frequency tables were used to characterize the study population. Time to first ANC visit was estimated using the Kaplan-Meier (K-M) method. The log-rank test was applied to compare the survival time difference between groups of categorical variables with the outcome of interest. In any applied set, survival data can be fitted using Cox Proportional Hazard [19], Accelerated Failure Time [19], and parametric shared frailty models [20]. Univariate and multivariate analyses were performed and all significant variables in univariate analyses (*p* < 0.05) were included in all multivariable analyses of the AFT shared frailty model and the best model was selected using AIC and BIC criteria. Data were entered and cleaned using SPSS-22 and analyzed using STATA-14.

### The Cox proportional hazard model

The Cox proportional model is proposed by [19] which is a semi-parametric model for the hazard function that allows the addition of covariates while keeping the baseline hazards unspecified and can take only positive values. This model gives an expression for the hazard at time t for an individual with a given specification of a set of explanatory variables denoted by X and it is generally given by:

1 $$ h\left(t,X,\beta \right)={h}_0(t)\exp \left( X\beta \right) $$

Where *h*_0_(*t*) is the baseline hazard function at time t, X is the vector of values of the explanatory variables and *β* = (*β*1, *β*2, …, *βk*) is the vector of unknown regression parameters that are assumed to be the same for all individuals in the study, which measures the influence of the covariate on the survival experience.

### Accelerated failure time model

In accelerated failure time models we assume that the effect of the covariates will be a multiplication of the expected survival time. A general formulation for the AFT hazard for an individual I with covariates P is summarized in vector 푿 [21].

2 $$ {h}_i(t)={e}^{-\eta i}{h}_o\left(t/{e}^{-\eta i}\right) $$

Where *ηi* = *a* ' *X* = *a*_1_*x*_1*i*_ + *a*_2_*x*_2*i*_ + ……. .  + *a*_*p*_*x*_*pi*_ is the linear component of the model in which 푥_ji_ is the value of 푗^th^ explanatory variable 푿_j_ for the i^th^ individual and exp − (*a*_1_*x*_1*i*_ + *a*_2_*x*_2*i*_ + ……. .  + *a*_*p*_*x*_*pi*_) is acceleration factor. Where *a*_1_, *a*_2_, …… . , *a*_*p*_ are the unknown regression coefficients of the explanatory variables *x*_1_, *x*_2_, …… . , *x*_*p*_.

The corresponding survivor function will be

3 $$ {S}_i(t){S}_o\left(t/{e}^{-\eta i}\right) $$

Where *s*_*o*_(*t*) the baseline survival function.

### Shared frailty models

Multivariate or shared frailty model is a conditional independence model in which frailty is common to all subjects in a cluster. The concept of frailty provides a suitable way to introduce random effects in the model to account for association and unobserved heterogeneity. In its simplest form, frailty is an unobserved random factor that modifies multiplicatively the hazard function of an individual or cluster of individuals [18]. introduced the term frailty and [22] promoted the model by its application to the multivariate situation on chronic disease incidence in families. The multivariate frailty model is an extension of the univariate frailty model which allows the individuals in the same cluster to share the same frailty value. When frailty is shared, dependence between individuals who share frailties is generated.

Let us have j observations and i subgroups. Each subgroup consists of n_i_ observations and $\sum \limits_{i=1}^r ni=n,$ where n is the total sample size. The hazard rate for the j^th^ individual in the i^th^ subgroup is given by:

4 $$ {h}_{ij}(t)={h}_0(t)\exp \left({X}_{ij}^{\prime}\beta +{Z}_i\right),i=1,2,\dots, r\ \mathrm{and}\ j=1,2,\dots, {n}_i $$

Here frailty Z is the random variable varying over the population decreases (Z < 1) or increases (Z > 1) the individual risk.

If the proportional hazards assumption does not satisfy, the accelerated failure time frailty model can be used.

5 $$ {h}_{ij}(t)={h}_0(t)\left(\exp \left({X}_{ij}^{\prime}\beta +{Z}_i\right)\right)\exp \left({X}_{ik}^{\prime }+{Z}_i\right) $$

### AFT shared frailty model

The AFT shared frailty model is an appropriate choice for multivariate clustered survival time data, especially when observations within a cluster share common unobservable frailty. It explicitly takes into account the possible correlation among failure times.

Suppose log*T*_*ij*_ be the logarithm of the survival time of the *j*^*th*^ pregnant woman in the *i*^*th*^ region, (*j* = 1, 2, …, *n*_*i*_ *and i* = 1, 2, …., 11), and *X*_*ij*_ be the vector of covariates associated with this individual. Then the shared AFT frailty model is given by:

6 $$ \log {T}_{ij}=\mu +x\hbox{'}{}_{ij}\beta +{Z}_i+\sigma {\in}_{ij} $$

Where β is the vector of unknown regression coefficients μ is the intercept parameter, σ is the scale parameter, the ∈_ij_’s are independent identically distributed random errors, and the Zi’s are the cluster-specific random effects which are assumed to be i.i.d random variable with density function f (zi). Here we have assumed that the shared frailty (random) effect Zi following gamma and inverse Gaussian distribution with mean zero and variance θ, as defined in the density function in Eqs. (4) and (5) respectively.

One important problem in the area of frailty models is the choice of the frailty distribution. Various studies have been done on the choice of distribution of frailty random variables. While some authors use continuous distributions such as Gamma [18, 22], inverse Gaussian [23, 24], log-normal [25] and positive stable [26]. However, the Gamma and Inverse Gaussian distribution are the most common and widely used in literature for determining the frailty effect, which acts multiplicatively on the baseline hazard [27] and [23].

7 $$ {f}_z(Z)=\frac{z^{\frac{1-\theta }{\theta }}\exp \left(\frac{-z}{\theta}\right)}{\theta^{\frac{1}{\theta }}\Gamma \frac{1}{\theta }},\theta >0 $$

8 $$ f(z)=\frac{1}{\sqrt{2\pi }}{z}^{\raisebox{1ex}{$-3$}\!\left/ \!\raisebox{-1ex}{$2$}\right.}\exp \left(\frac{-1}{2\theta z}{\left(z-1\right)}^2\right) $$

Where θ > 0, indicates the presence of heterogeneity. So, the large values of θ reflect a greater degree of heterogeneity among regions of pregnant women and a stronger association within regions. In these models, frailty could be considered as an unobserved covariate that is additive on the log failure time scale and describes some reduced or increased event times for different clusters. All observations within a cluster share a common unobserved random effect. Now the conditional survivor function and hazard function for the j^th^ individual of i^th^ cluster is written as:

9 $$ {S}_{ij}\left(t/{z}_i\right)={S}_0\left({\in}_{ij}/{Z}_i\right) $$

10 $$ {h}_{ij}\left(t/{z}_i\right)=\frac{1}{\sigma {t}_{ij}}{h}_0\left({\in}_{ij}/{Z}_i\right) $$

From equation [9], we have ${\in_i}_j=\frac{\mathit{\log}{t}_{ij}-\mu -X{\prime}_{ij}\beta -{Z}_i}{\sigma .}$

Where *S*_0_(.) and *h*_0_(.) are the survivor and hazard function of ∈_*ij*_ respectively, and β is a vector of fixed effects associated with a vector of covariates *X*_*ij*_ measured on the *j*^*ih*^ individual in the *i*^*th*^ cluster.

The associations within group members (regions) are measured by Kendall’s, for gamma frailty distribution is given by: -

$$ \Gamma =\frac{\theta }{\theta +2}\in \left(0,1\right) $$

## Results

### Descriptive statistics

The descriptive summary of covariates is given in Table 2 shows a total of 4328 of women who got pregnant during 5 years’ survey were included in this study from nine regions and two administrative cities of whom, 1210 (28%) received first ANC visit (events) and 3118(72%) did not receive first ANC visit (right censored). Among pregnant women included in the study, the highest number was from SNNP 608(14%) while the lowest numbers were from Afar, 275(6.4%) followed by Gambela 288 (6.6%), and Dire Dawa 291(6.7%). On the other hand, 2176(50.1%) women had no education and among these only 499(11.5%) were following ANC visit (event). In the educational status of husbands, 1599(36.9%) were had no education, 1561(36.1%) had attended primary education and the remaining 1168(27%) were attending secondary and above education level. On the residence of pregnant women, 3094 (71.5%) were from a rural setting, the 710(16.4%) had ANC visits. In the wealth index of pregnant women, the majority were rich 1983(45.8%) followed by poor, 1691 (39.1%), and of these 1294(29.9%) and 1346(17%) had no ANC visit (censored) respectively. Furthermore, among pregnant mothers included in the analysis, 2482 (57%) had a problem with media access; while the remaining 1846(43%) reported media exposure was not their problem. But 1903(44%) of those who had a problem with media access had no ANC visit, whereas 1215(28.1%) of those whose reported media exposure was not their problem had no ANC visit. Regarding decisions on respondent’s health care, it was found that 797 (18.4%) decided by themselves, 2817(65.1%) decided together with their husband/partner while 714(16.5%) decided by husband/Partner and other persons and out of these, 558(12.9%), 2014(46.5%) and 2014(46.5%) had no ANC visit respectively. On marital status, the majority of pregnant women 4271(98.6%) were married and of these, 3075(71%) had no ANC visit and the remaining 57(1.4%) were separated (Table 2).

**Table 2:** Socio-demographic and obstetric characteristics of pregnant women in Ethiopia, EDHS 2016

Variable	Categories	Time to first ANC visit	
		Event No (%)	Censored No (%)
Age	15–24	324 (7.5%)	830 (19%)
	25–34	715 (16.5%)	1757 (40.5%)
	35–49	171 (4%)	531 (12.5%)
Mother education level	No education	499 (11.5%)	1677 (38.7%)
	Primary	406 (9.3%)	995 (23%)
	Secondary and above	305 (7.1%)	446 (10.4%)
Residence	Urban	500 (11.6%)	734 (17%)
	Rural	710 (16.4%)	2384 (55%)
Wealth index	Poor	345 (8%)	1346 (17%)
	Middle	176 (4%)	478 (31.1%)
	Richer	689((16%)	1294 (29.9%)
Marital status	Separated	14 (0.3%)	43 (1%)
	Married	1196 (27.6%)	3075 (71%)
Mother occupation currently	No	805 (18.6%)	2160 (50%)
	Yes	405 (9.4)	958 (22%)
Husband education level	No education	366 (8.4%)	1233 (28.5%)
	Primary	396 (9.1%)	1165 (27%)
	Secondary and above	448 (10.4%)	720 (16.6%)
Parity	3 or less	819 (19%)	1816 (42%)
	4 and above	391 (9%)	1302 (30.1%)
Sex of household head	Male	974 (22.5%)	2598 (60%)
	Female	236 (5.4%)	520 (12%)
A person who usually decides on respondent’s health care	Respondent alone	239 (5.5%)	558 (12.9%)
	Respondent and husband/partner	803 (18.6%)	2014 (46.5%)
	Respondent and another person	168 (3.9%)	546 (12.6%)
Wanted pregnancy	Then	995 (23%)	2506 (60%)
	Later	170 (3.9%)	448 (10.4%)
	No more	45 (1.04%)	164 (3.8%)
Husband occupation	Not working	101 (2.3%)	231 (5.3%)
	Agriculture	501 (11.6%)	1549 (35.8%)
	Non-agricultural	608 (14%)	1338 (30.9%)
Religion	Orthodox	497 (11.5%)	1158 (26.7%)
	Muslin	504 (11.6%)	1308 (30%)
	Protestant	191 (4.4%)	609 (14.1%)
	Other	18 (0.4%)	43 (0.9%)
Media exposure	No	579 (13.4%)	1903 (44%)
	Yes	631 (14.6%)	1215 (28.1%)

### Non-parametric survival analysis

#### The Kaplan-Meier estimate of time-to-first ANC visit

**Fig. 2:**
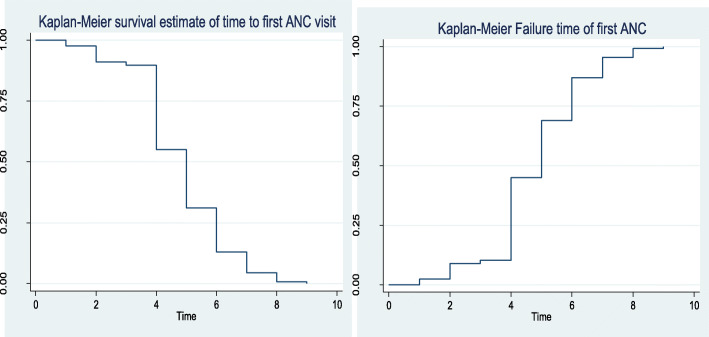
The K-M plots of Survival and hazard functions of time to first ANC visit among pregnant women in Ethiopia

Non-parametric survival analysis (K-M) is used to visualize the survival time-to-first ANC visit of pregnant women in Ethiopia under different covariates. It also provides information on the shape of the survival and hazard function of the ANC data set. The survival plot in Fig. 2 sharply decreased first and slowly decline at later times. This implies the probability of not starting an ANC visit is higher at early gestational age and tends to sharply decrease later as gestational age increased. Furthermore, the median time of the first ANC visit for pregnant women in Ethiopia was at the 5th month.

#### Comparison of a place of residence in terms of survival time to first ANC visit

**Fig. 3:**
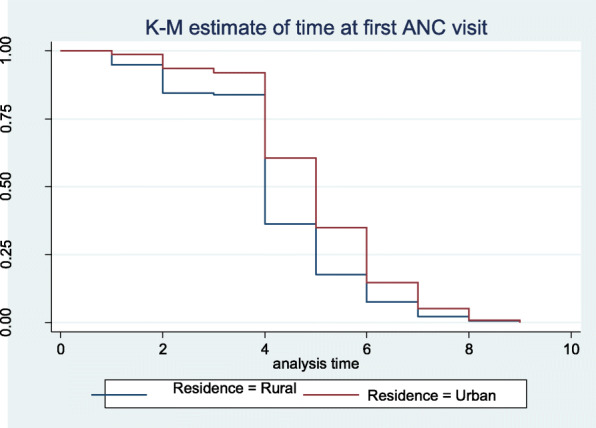
Survival time to first ANC visit among pregnant women by place of residence

Kaplan Meier graphs are used to depict the waiting time to first ANC visit of pregnant women for different covariates (mother’s characteristics). Figure 3 shows that pregnant mother from the rural area started first ANC visit late compared to those from an urban area or the probability of not starting ANC visit were higher through gestational age for pregnant women from rural compared to an urban residence. In addition, the log-rank test in Table 3 shows there is a statistically significant difference between them in terms of waiting time to first ANC visit (*p*-value< 0.001).

**Table 3:** Comparison of survival time, for first antenatal care visit (in months) among pregnant women in Ethiopia EDHS, 2016

Variable	Categories	Time to first ANC visit		Log-Rank test	*P*-value
		Event	Censored		
Age	15–24	324	830	8.463	.015
	25–34	715	1757		
	35–49	171	531		
Mother education level	No education	499	1677	135.804	.000
	Primary	406	995		
	Secondary and above	305	446		
Residence	Urban	500	734	202.167	.000
	Rural	710	2384		
Wealth index	Poor	345	734	123.590	.000
	Middle	176	2384		
	Richer	689	3118		
Marital status	Separated	14	43	.123	.726
	Married	1196	3075		
Mother occupation currently	No	805	2160	4.138	.042
	Yes	405	958		
Husband education level	No education	366	1233	129.314	.000
	Primary	396	1165		
	Secondary and above	448	720		
Parity	3 or less	819	1816	46.101	.000
	4 and above	391	1302		
Sex of household head	Male	974	2598	7.061	.008
	Female	236	520		
A person who usually decides on respondent’s health care	Respondent alone	239	558	10.721	.005
	Respondent and husband/partner	803	2014		
	Respondent and another person	168	546		
Wanted pregnancy	Then	995	2506	7.248	.027
	Later	170	448		
	No more	45	164		
Husband occupation	Not working	101	231	7.248	.000
	Agriculture	501	1549		
	Non-agricultural	608	1338		
Religion	Orthodox	497	1158	13.705	.003
	Muslin	504	1308		
	Protestant	191	609		
	Other	18	43		
Media exposure	No	579	1903	89.015	.000
	Yes	631	1215		

#### Comparisons of the different covariate in terms of survival time to first ANC visit

A formal test was carried out using the log-rank to compare the difference between each categorical variable. The log-rank test result (Table 3) shows that there is no significant difference in the ‘survival’ experience of different categories for marital status (*p* = 0.726). Similarly, the log-rank test performed for different covariates indicate there is statistically significant difference in survival experience among age group (*p* < 0.001), mother education level (*p* < 0.001), residence (*p* < 0.001), religion (*p* < 0.001), wealth index (< 0.001), education of husband (*p* < 0.001), husband occupation (*p* < 0.001), parity (*p* < 0.001), media exposure (*p* < 0.001) and wanted pregnancy (*p* = < 0.001).

#### Test of proportional hazard assumption by Schoenfeld residual

The proportionality of the Cox proportional hazard model can be tested using rho statistic, *p*-value, and Scaled Schoenfeld residuals. The large value of rho showed a strong correlation between residuals and time because of this there is the existence of a systematic pattern on the graph which showed that the proportional hazard assumption is not satisfied. The *p*-value of the rho statistic is less than 5% for a given covariate indicates the rejection of the null hypothesis of the proportionality of the Cox proportional hazard model.

#### Accelerated failure time model result

Since the proportional hazards assumption was not satisfied, the accelerated failure time model is an alternative model for the analysis of this data. We fitted the data using an accelerated failure time model with Weibull, Lognormal, and Log-logistic as a baseline distribution with and without different frailty distributions. The best model is the one with the lowest value of AIC and BIC. AIC and BIC values of Weibull Inverse Gaussian shared frailty were the smallest (5190.44 and 5356.138) which is presented in Table 4. Therefore, Weibull Inverse Gaussian shared frailty was the best model to describe the given pregnant women ANC visit data Table 5.

**Table 4:** Assumption of proportional hazard model

Covariate	Categories	Rho	Chis-q	*P*-value
Marital Status	Separated	Ref		
	Married	0.02495	1.99	0.1587
*Residence*	Urban	Ref		
	Rural	0.06345	14.15	0.0002
Mother Occupation	No	Ref		
	Yes	−0.01047	0.36	0.5474
*Mother education*	No education	Ref		
	Primary	−0.00075	0.002	0.9663
	Secondary and above	−0.01402	0.64	0.4251
*Wealth index*	Poor	Ref		
	Middle	0.02432	1.95	0.2899
	Rich	0.01852	1.12	
*Wanted pregnancy*	Then	Ref		
	Later	0.00813	0.21	0.6446
	No more	0.02967	2.82	0.09332
*Sex of household*	Male	Ref		
	Female	0.01878	1.16	0.2824
*Husband Occupation*	Not working	Ref		
	Agriculture	0.00544	0.10	0.7536
	Non agricultural	−0.01727	0.94	0.3314
*Religion*	Orthodox	Ref		
	Muslim	−0.00831	0.37	0.5419
	Protestant	0.04012	8.44	0.0037
	Other	0.01554	0.85	0.3575
*Parity*	3 and bellow	Ref		
	4 and above	0.00346	0.15	0.54
*Husband education level*	No education	Ref		
	Primary	−0.23567	1.89	0.0034
	Secondary and above	0.89302	0.49	0.6702
*The person decided on the respondent’s health care*	Respondent alone	Ref		
	Respondent alone and Husband/partner	0.00813	0.08	0.7823
	Respondent/partner and other person	0.00813	0.21	0.6467
*Age*	15–24	Ref		
	25–34	0.01554	0.79	0.3752
	35–49	0.00450	0.07	0.7948
*Media exposure*	No	Ref		
	Yes	−0.01413	0.62	0.4307
Global test			79.04	0.0000

**Table 5:** Comparison of gamma and Inverse Gaussian shared frailty model with different baseline distributions

Information criteria	Models		Frailty distributions	
		No frailty	Gamma shared frailty	Inverse Gaussian shared frailty
AIC	Exponential	6338.547	6260.358	6259.905
	Weibull	5782.508	5207.318	5190.444
	Lognormal	5668.414	5446.669	5425.664
	Log logistic	5908.365	5789.093	5788.304
BIC	Exponential	6491.496	6419.679	6419.226
	Weibull	5941.83	5373.013	5356.138
	Lognormal	5827.736	5612.364	5591.358
	Log logistic	6067.686	5954.787	5953.998

Univariable and multivariable analysis was performed to select variables to be included in the model. In univariable analysis at a 25% level of significance except for age, all variables were significant. The variables significant in univariable analysis entered to multivariable analysis.

#### Weibull Inverse Gaussian shared frailty model result

The frailty in this model is assumed to follow Inverse Gaussian distribution with mean 1 and variance equal to theta (θ). The result of the Weibull- Inverse Gaussian shared frailty model is given below in Table 5. From this result, the frailty term θ = 0.180 indicates that there is heterogeneity between regions. A likelihood ratio test for the hypothesis θ = 0 indicates a chi-square value of 122.06 with one degree of freedom resulting in a highly significant *p*-value of 0.005. This implied that the frailty component had a significant contribution to the model. Kendall’s tau (τ) is used to measure the dependence within the clusters. From the results of this study, the values of Kendall’s tau (τ) for the Weibull- Inverse Gaussian frailty is 0.432. The estimated value of the shape parameter in this selected model was *P* = 1.843. This value is greater than unity these indicate the shape of hazard functions is increases up as time increase (Table 6).

**Table 6:** Weibull Inverse Gaussian shared frailty model results

Variable	Coef.	SE	*Φ*	95% CI for *ϕ*	*P*-value
Residence					
Rural	0.175	0.063	1.191	[1.073, 1.322]	0.001
Marital Status					
Married	−0.209	0.121	0.811	[0.606, 1.087]	0.161
Mother occupation					
Yes	−0.042	0.034	0.959	[0.895, 1.027]	0.232
*Wanted pregnancy*					
*Latter*	0.066	0.049	1.069	[0.976, 1.169]	0.150
*No more*	0.192	0.103	1.212	[1.026, 1.431]	0.670
Media exposure					
Yes	−0.077	0.037	0.926	[0.856, 1.09]	0.005
*Sex of household*					
Female	−0.028	0.041	0.972	[0.895, 1.056]	0.503
Religion					
Muslim	−0.029	0.045	0.971	[0.888, 1.063]	0.527
Protestant	0.052	0.063	1.053	[0.937, 1.184]	0.383
Other	−0.172	0.114	0.842	[0.645, 1.099]	0.206
Wealth index					
Middle	−0.174	0.044	0.840	[0.759, 0.931]	0.001
Rich	−0.139	0.044	0.870	[0.788, 0.961]	0.006
Mothers education					
Primary	−0.077	0.040	0.926	[0.852, 1.007]	0.073
Secondary and above	−0.158	0.049	0.854	[0.762, 0.956]	0.006
*Husband Occupation*					
*Agriculture*	0.039	0.067	1.040	[0.917, 1.180]	0.541
*Non Agricultural*	0.150	0.073	1.162	[1.027, 1.313]	0.017
Parity					
4 and above	0.055	0.045	1.056	[0.971, 1.149]	0.204
Husband education level					
Primary	−0.050	0.042	0.951	[0.872, 1.036]	0.251
Secondary and above	−0.183	0.044	0.833	[0.750, 0.925]	0.001
*Person decided on respondent’s health care*					
Respondent alone and Husband/partner	0.062	0.045	1.064	[0.979, 1.156]	0.146
Respondent/partner and other person	0.071	0.061	1.074	[0.961, 1.200]	0.209
Theta ( )	0.180				
P = 1.843, 1/*p* = 0.543, λ = 9.933, θ = 0.180, τ = 0.432					

LR test of theta = 0: chibar2 (01) = 122.06, Prob > = chibar2 = 0.000

*Coef* coefficient of parameters, *Std. Err.* standard error of parameters, *τ* Kendall’s tau, *ϕ* acceleration factor, *95% CI* 95% confidence interval for acceleration factor, *P* shape parameter, *λ* scale parameter, θ variance of the random effect, *prob* probability, *chibar2* Chi-square

#### Interpretation of Weibull-Inverse Gaussian shared frailty model results

From Table 5 the confidence intervals of the acceleration factor for covariates that do not include one are significant at 5% level significance.

The covariate residence was statistically determined for the time to first ANC visit. The acceleration factor and 95% confidence interval of residence for a group of rural were 1.191 (1.073, 1.322) when compared to urban group (as reference category) respectively. This indicates rural women have prolonged time-to start first ANC visit than urban women. The acceleration factor and its 95% CI for pregnant women who have used media exposure were 0.926 and (0.856, 1.09) respectively. This shows 7.4% less survival time for time to first ANC visit who has access to media than who did not get it. The acceleration factor and 95% confidence interval for wealth index of pregnant women for a group middle and rich were 0.840 (0.759, 0.931) and 0.87(0.788, 0.961) respectively using the poorest as a reference category. This indicates that for middle and richest groups started ANC earlier than the reference group at a 5% level of significance. The acceleration factor and 95% confidence interval for pregnant women who attend secondary and higher education levels are 0.854 and (0.762, 0.956) respectively. This indicates educated women have shortened time-to start first ANC visit than uneducated pregnant women at a 5% level of significance. The estimated coefficient of the parameter for husband occupation who were working in non-agricultural activities was 0.150. The sign of the coefficient was positive which implies an increase in the log of survival time and hence, elongated expected duration of time to first ANC visit than whose husband is not working (reference group. Moreover, the acceleration factor and 95% confidence interval of husband education level for a group secondary and above was 0.833 and (0.750, 0.925) times no education (reference group). This indicates a pregnant woman whose husband is not educated has prolonged time to start the first ANC visit than whose husband was educated (reference group) at a 5% level of significance.

### Model diagnostics

#### Cox-Snell residual plots

**Fig. 4:**
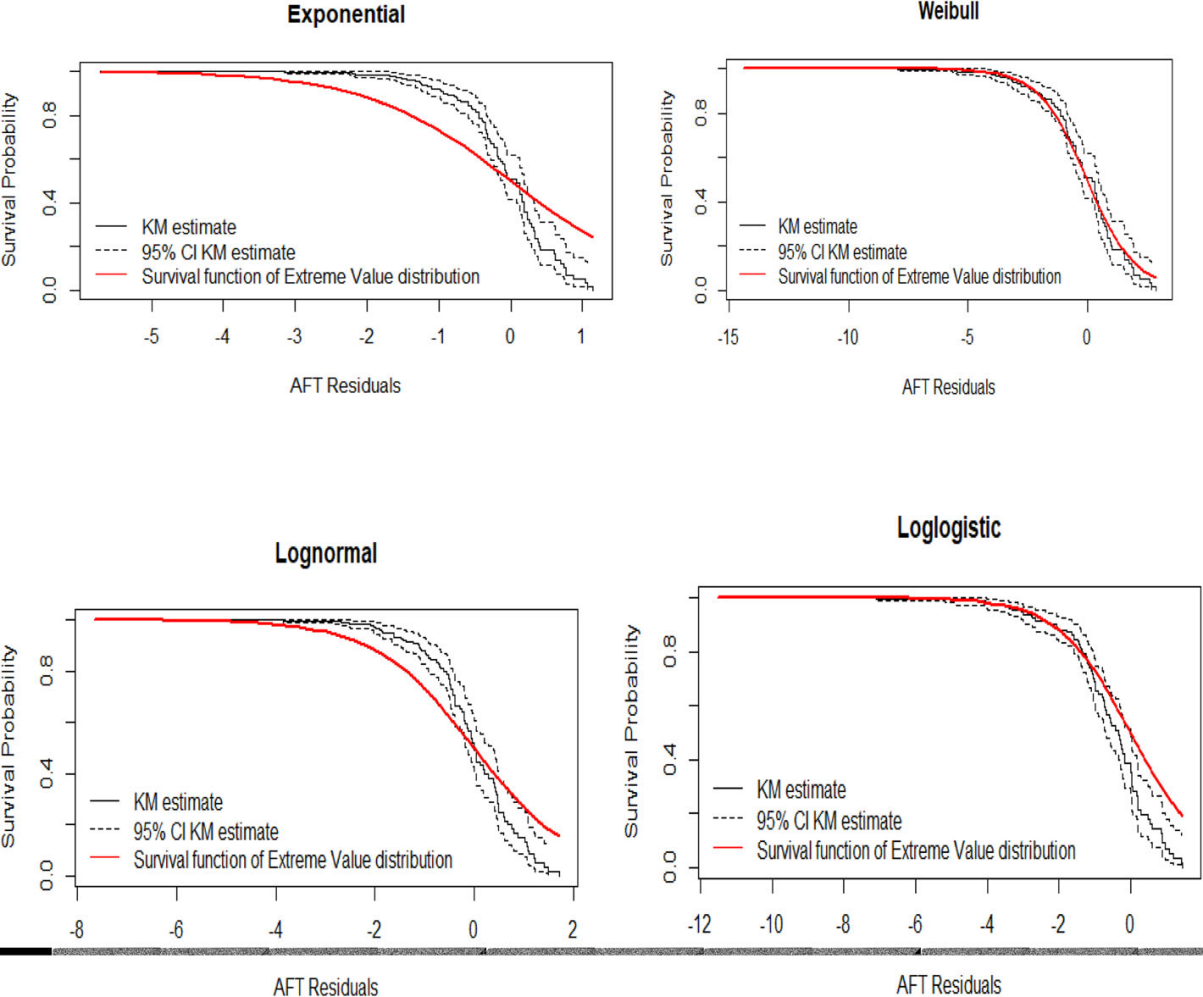
Cox-Snell residual plots for exponential, log-normal, log-logistic, and Weibull AFT models

The Cox-Snell residuals method can be applied to any parametric model. The Cox-Snell residuals are one way to investigate how well the model fits the data. The diagnostic is based on Cox-Snell residuals with the 95% point-wise CI for the Kaplan-Meier estimate of the Cox-Snell residuals along the red line. Since the exponential, lognormal and log-logistic distributions become be below and above the confidence intervals, but for Weibull baseline distribution the line is more in touch with the Kaplan-Meier estimate line and completely within the confidence intervals therefore the Weibull distribution provides a good fit to the data (Fig. 4).

## Discussion

The main objective of this study was to identify determinant factors for time-to-first ANC visit using AFT shared frailty models by considering different baseline distributions to investigate a model that is better in predicting time to first ANC visit in Ethiopia. The comparison of distributions of the models was done using AIC criteria, where a model minimum AIC was accepted [28]. All significant variables in univariate analyses were included in all multivariable analyses of the AFT model and the best model was selected using AIC criteria. Weibull AFT model was best based on AIC and BIC value from Table 4. After analyzing the given data set by using the Weibull AFT model, AFT shared frailty models by assuming gamma distribution and Inverse Gaussian for the frailty term were fitted by considering Exponential, Weibull, log-logistic, and log-normal baseline distributions. Weibull Inverse Gaussian shared frailty model was selected based on AIC and BIC values. The aim of the frailty model is not only to account for heterogeneity subjects among different regions but also to measure the dependence or correlation within the same region. The clustering effect was significant (*p*-value = 0.000) in Weibull-Inverse Gaussian shared frailty model. This showed that there was heterogeneity between the regions on the time to first ANC visit of pregnant women in Ethiopia.

The result of the Weibull Inverse Gaussian shared frailty model shows residence, media exposure, wealth index, mother education level, husband occupation, and husband education level are significantly associated with time to first ANC visit of pregnant women at 5% of the significance level. This is consistent with a study conducted in [12, 13, 29].

Based on the given dataset place of residence of the woman was the factor that affects the survival time of first antenatal care during the gestational age. As it is indicated in Table 5, the acceleration indicates rural women have prolonged time-to-start first ANC visits than urban women. Similar studies also publicize comparable findings [4, 10]. This later initiation of ANC among rural women could be due to better access to health facilities in urban areas than in rural areas. In addition, Media exposure was positively and significantly related with time to the first ANC visit, and this is consistent with previous studies [4, 11]. The presence of media access indirectly indicates the relatively wealthy household, urban residency, better education level, and easy access to healthcare services. The sums of these factors also empower women to have the autonomy to engage in healthcare services that improve the health of the women and unborn baby including timely commencement of ANC [4]. Moreover, the study also revealed that women from the richest and middle wealth index households were started first ANC earlier than women from poor households. This is similar to reports by [11, 30], suggesting that women from the richest wealth index were more likely to initiate ANC at an earlier gestational age.

The other important variable in our study is education level, educated women and women having educated husbands are associated with early antenatal care visits. This finding was in agreement with findings in [10, 13, 30]. Educated husbands may have positive maternal and child health knowledge to encourage their wives to initiate antenatal care at the early age of the pregnancy. One reason could be also those women have educated husband also will be educated themselves.

## Conclusion

To identify the factors associated with time-to-first ANC visits, different AFT models with associated different shared frailty distributions were applied. Among these using AIC and BIC criteria, Weibull Inverse Gaussian shared frailty model was better fitted to the time-to-first ANC visit dataset than other and AFT shared frailty models. There was a frailty (clustering) effect on the time-to first ANC visit that arises due to differences in the distribution of the timing of the first ANC visit among regions of Ethiopia. This indicates the presence of heterogeneity and necessitates the frailty models. This heterogeneity could be arising due to environmental, socio-cultural differences in utilization of health care services and variation in accessing health services across the regions of Ethiopia. In this study the major factors for time to first ANC visit identified were, residence, media exposure, wealth index, educational levels of mother and husband and husband occupation are statistically significant at 5% level of significance. As a result, all responsible entities should take decisive and appropriate action to prevent negative fetomaternal outcomes as a result of delayed ANC visits. To raise community understanding of the timing of the initial ANC visit, health care practitioners and community health workers should deliver health education. Finally, the Ethiopian Ministry of Health and Regional Health Bureau must devise a strategy to increase the number of women who use early ANC follow-up, as well as construct health care facilities closer to people, particularly for women who live in remote areas. Generally, It is essential that maternal and child health policies and strategies better target women’s development and design and implement interventions aimed at increasing the timely activation of prenatal care by pregnant women. The researchers also recommend using more powerful designs (such as cohorts) for the research to establish timeliness and reduce death.

## Data Availability

The dataset and supporting materials will be obtained based on a request from the corresponding author on a reasonable request.

## Abbreviations

ANC: Antenatal care
AFT: Accelerated failure time
AIC: Akaike’s information criterion
BIC: Bayesian information criterion
CSA: Central Statistical Agency
EDHS: Ethiopia Demographic and Health Survey
PH: Proportional Hazard
WHO: World Health Organization
